# Editorial: Early cervical cancer: laparotomic vs minimally invasive surgery and fertility-sparing possible strategies

**DOI:** 10.3389/fmed.2024.1415558

**Published:** 2024-05-03

**Authors:** Violante Di Donato, Giorgio Bogani, Antonio Simone Laganà, Andrea Giannini

**Affiliations:** ^1^Department of Maternal and Child Health and Urological Sciences, Sapienza University of Rome, Rome, Italy; ^2^Department of Gynecologic Oncology, IRCCS National Cancer Institute, Milan, Italy; ^3^Unit of Obstetrics and Gynecology, “Paolo Giaccone” Hospital, Department of Health Promotion, Mother and Child Care, Internal Medicine and Medical Specialties (PROMISE), University of Palermo, Palermo, Italy; ^4^Unit of Gynecology, Sant'Andrea Hospital, Department of Surgical and Medical Sciences and Translational Medicine, Sapienza University of Rome, Rome, Italy

**Keywords:** cervical cancer, minimally invasive surgery, laparotomic surgery, fertility-sparing, gynecologic surgery

Cervical cancer (CC) is the third most common malignancy in terms of both incidence and mortality among women, with ~604,000 new cases and 342,000 deaths reported in 2020 (1). The gold standard treatment for patients with early-stage CC (FIGO stages IA2-IB1) is radical hysterectomy (RH) (2, 3). For years, laparotomy has been frequently performed, achieving high cure rates. Although several studies have shown the superiority of the open surgical approach in terms of overall survival (OS) and disease-free survival (DFS), showing higher recurrence rates with the use of minimally invasive surgery (MIS), growing evidence supports the adoption of the MIS, which includes laparoscopic and robotic techniques, for the treatment of early-stage CC (4–6). These surgical approaches demonstrate benefits despite suboptimal oncologic outcomes (7–9). In addition, a fertility-sparing strategy should be considered for patients expressing a desire for future childbearing. The procedures of conization, simple trachelectomy, and radical trachelectomy represent the fertility-sparing approaches (2). However, the use of minimally invasive techniques is debated and continuously questioned, especially for cervical cancers (4, 5, 7).

The main aim of this Research Topic was to focus on oncologic and surgical outcomes by comparing laparotomic hysterectomy with minimally invasive hysterectomy for early-stage CC patients.

Four high-quality papers were published on this Research Topic: two original research, one review, and one ongoing randomized controlled trial.

Given the controversy in identifying the optimal surgical technique, conducting randomized trials is essential to reach a shared consensus. Tang et al. are among the investigators who are implementing a three-arm randomized trial in which participants will be randomly assigned to one of the following three groups: conventional laparoscopic RH, laparoscopic RH without the use of gas, or abdominal RH. For a sample of ~500 patients, 2-year DFS and 5-year OS will be evaluated. Surgical outcomes will also be examined, including duration of surgery, intraoperative blood loss, surgical complications, and patients' quality of life. Analysis of the results obtained will provide important information and help improve the clinical management and counseling of patients with this disease, guiding the selection of the optimal surgical technique to maximize oncologic outcomes and improve the overall quality of care.

One of the main advantages of minimally invasive surgery lies in the reduction of the postoperative stay and the early discharge of patients undergoing surgery. In Liu et al. review, factors that might influence the daily hospitalization of patients undergoing hysterectomy were examined. Twenty-nine studies were included, involving a total of 218,192 patients undergoing minimally invasive surgery for both benign and malignant conditions.

What emerged from the analysis highlights that factors including increasing age, body mass index, and the presence of comorbidities such as diabetes, pulmonary, cardiac, or cardiovascular disease may influence the possibility of same-day discharge. In addition, the type of surgical procedure performed was found to be a determining factor; in fact, RH was unfavorable for same-day discharge, as was prolonged operative time. Gynecological affections, both malignant and benign, showed similar same-day discharge rates. However, the time of surgery start time and BMI have a greater influence on the likelihood of same-day discharge in malignant conditions than in non-malignant conditions.

The primary objective of surgery is to achieve optimal radicality because the type of surgery can have a significant impact on OS and DFS. Wang, Liu, Duan, Li, Su et al. conducted a retrospective study of 426 patients to evaluate the survival outcomes associated with Querleu-Morrow type B RH and type C RH in patients with early-stage CC. The results showed that there were no significant differences in 5-year OS and DFS between the group undergoing type B RH and the group undergoing type C RH (OS: 87.8 vs. 89.4%, *p* = 0.814; DFS: 84.9 vs. 85.6%, *p* = 0.898). These results suggest that both surgical procedures can be considered viable options for patients with early-stage CC, allowing surgeons to adopt a personalized strategy based on specific patient characteristics and surgical preferences.

An individualized surgical approach could contribute to the reduction of surgical morbidity. Patient analysis using advanced methodologies such as computed tomography or magnetic resonance imaging, resulting in 3-D models, would allow individualized planning of paratumoral tissue resection in patients with CC. Wang, Liu, Duan, Li, Li et al., through a retrospective analysis conducted on 374 patients undergoing RH, acquired instrumental imaging data for 3D modeling. It was found that the greatest survival benefits were observed when the paratumoral resection range reached 32.35 mm in patients with stromal invasion < 1/2 the depth. To achieve favorable oncological outcomes in patients exhibiting stromal invasion exceeding half the myometrial depth, a minimum paratumoral resection margin of 32.35 mm was required. This assessment could allow more accurate customization of the type of cardinal ligament resection in CC patients.

In conclusion, the use of MIS techniques remains debated, and many challenges remain regarding the application of MIS for the treatment of gynecological cancers, particularly cervical cancers, leaving the need for randomized trials to establish its role and the need for further investigation.

We hope that this Research Topic will spark the reader's interest, inspiring new ideas for future research.

## Author contributions

VD: Conceptualization, Writing – original draft, Writing – review & editing. GB: Writing – original draft, Writing – review & editing. AL: Writing – original draft, Writing – review & editing. AG: Conceptualization, Methodology, Validation, Visualization, Writing – original draft, Writing – review & editing.

## References

[1] Sung H Ferlay J Siegel RL Laversanne M Soerjomataram I Jemal A. Global cancer statistics 2020: GLOBOCAN estimates of incidence and mortality worldwide for 36 cancers in 185 countries. CA Cancer J Clin. (2021) 71:209–49. 10.3322/caac.21660 33538338

[2] Cibula D Raspollini MR Planchamp F Centeno C Chargari C Felix A. ESGO/ESTRO/ESP guidelines for the management of patients with cervical cancer - update 2023. Int J Gynecol Cancer. (2023) 33:649–66. 10.1136/ijgc-2023-004429 37127326 PMC10176411

[3] Giannini A D'Oria O Chiantera V Margioula-Siarkou C Di Donna MC Terzic S. Minimally invasive surgery for cervical cancer: should we look beyond squamous cell carcinoma? J Invest Surg. (2022) 35:1602–3. 10.1080/08941939.2022.2075495 35549629

[4] Ramirez PT Frumovitz M Pareja R Lopez A Vieira M Ribeiro R. Minimally invasive versus abdominal radical hysterectomy for cervical cancer. N Engl J Med. (2018) 379:1895–904. 10.1056/NEJMoa1806395 30380365

[5] Di Donato V Bogani G Casarin J Ghezzi F Malzoni M Falcone F. Ten-year outcomes following laparoscopic and open abdominal radical hysterectomy for ≪low-risk≫ early-stage cervical cancer: a propensity-score based analysis. Gynecol Oncol. (2023) 174:49–54. 10.1016/j.ygyno.2023.04.030 37149905

[6] Chiva L Zanagnolo V Querleu D Martin-Calvo N Arévalo-Serrano J Cápîlna ME. SUCCOR study: an international European cohort observational study comparing minimally invasive surgery versus open abdominal radical hysterectomy in patients with stage IB1 cervical cancer. Int J Gynecol Cancer. (2020) 30:1269–77. 10.1136/ijgc-2020-001506 32788262

[7] Pecorino B D'Agate MG Scibilia G Scollo P Giannini A Di Donna MC. Evaluation of surgical outcomes of abdominal radical hysterectomy and total laparoscopic radical hysterectomy for cervical cancer: a retrospective analysis of data collected before the LACC trial. Int J Environ Res Public Health. (2022) 19:13176. 10.3390/ijerph192013176 36293758 PMC9603513

[8] Sert BM Kristensen GB Kleppe A Dørum A. Long-term oncological outcomes and recurrence patterns in early-stage cervical cancer treated with minimally invasive versus abdominal radical hysterectomy: The Norwegian Radium Hospital experience. Gynecol Oncol. (2021) 162:284–91. 10.1016/j.ygyno.2021.05.028 34083029

[9] Bogani G Donato VD Scambia G Landoni F Ghezzi F Muzii L. Practice patterns and 90-day treatment-related morbidity in early-stage cervical cancer. Gynecol Oncol. (2022) 166:561–6. 10.1136/ijgc-2022-ESGO.4 35909005

